# A Case of Drug-induced Linear IgA Bullous Dermatosis

**DOI:** 10.7759/cureus.7175

**Published:** 2020-03-04

**Authors:** Sofanit A Dessie, Davinder Singh, Varun Dobariya, Derek Evans, Peimei He

**Affiliations:** 1 Internal Medicine, Marshall University, Joan C. Edwards School of Medicine, Huntington, USA; 2 Medicine, Cabell Huntington Hospital, Huntington, USA; 3 Internal Medicine, Marshall University, Huntington, USA

**Keywords:** linear iga bullous dermatosis, vancomycin, piperacillin-tazobactam

## Abstract

Linear immunoglobulin A (IgA) bullous dermatosis (LABD) presents as a rare autoimmune disease that can either occur spontaneously or induced by certain drugs, the most common of which is vancomycin. LABD is a subepidermal blistering disease that is diagnosed by detecting linear IgA bands along the basement membrane. We present a case of a 59-year-old man with worsening blistering skin rash who was treated with vancomycin and piperacillin-tazobactam for pneumonia.

## Introduction

Intravenous (IV) vancomycin and piperacillin-tazobactam are commonly administered antibiotics to provide a broad spectrum coverage of gram-positive organisms including methicillin-resistant Staphylococcus aureus (MRSA) and gram negatives respectively, frequently given as an empiric therapy for systemic infections including pneumonia [1,2]. Drug-induced linear immunoglobulin A (IgA) bullous dermatosis (LABD) is a rare autoimmune vesiculobullous disorder that affects an older population of adults (mean age of 66.5) [3]. The clinical presentation of LABD may be variable and mimic other blistering disorders such as bullous pemphigoid, cicatricial pemphigoid, dermatitis herpetiformis, erythema multiforme, and toxic epidermal necrolysis (TEN) [4]. In addition to the usual vesiculobullous presentation, vancomycin-induced LABD can also present with erythematous papules, urticarial lesions, eczematous patches, TEN, and erosions [5]. The uncommon nature and variable presentation of LABD makes diagnosis difficult and leads to a delayed recognition of the disease. We present a case where vancomycin and piperacillin-tazobactam were used for presumed pneumonia and likely resulted in drug-induced LABD leading to septic shock and death.

## Case presentation

A 59-year-old male nursing home resident with a past medical history of quadriplegia secondary to a remote ischemic stroke, chronic obstructive pulmonary disease, coronary artery disease, and chronic kidney disease initially presented to an outside hospital with increased cough and shortness of breath. He was admitted and treated with five days of IV vancomycin and piperacillin-tazobactam for presumed pneumonia. Two weeks after taking these antibiotics, he developed progressive, diffuse, tense rash with blisters that initially started over the scrotum and progressed to involve the oral mucosa, the extremities including palms and soles, chest, torso, and genital regions. He was reported to have persistent symptoms of fever, cough and shortness of breath for which he received another three days of the same antibiotic regimen for persistent pneumonia before he was transferred to our hospital, for management of worsening rash.

**Figure 1:**
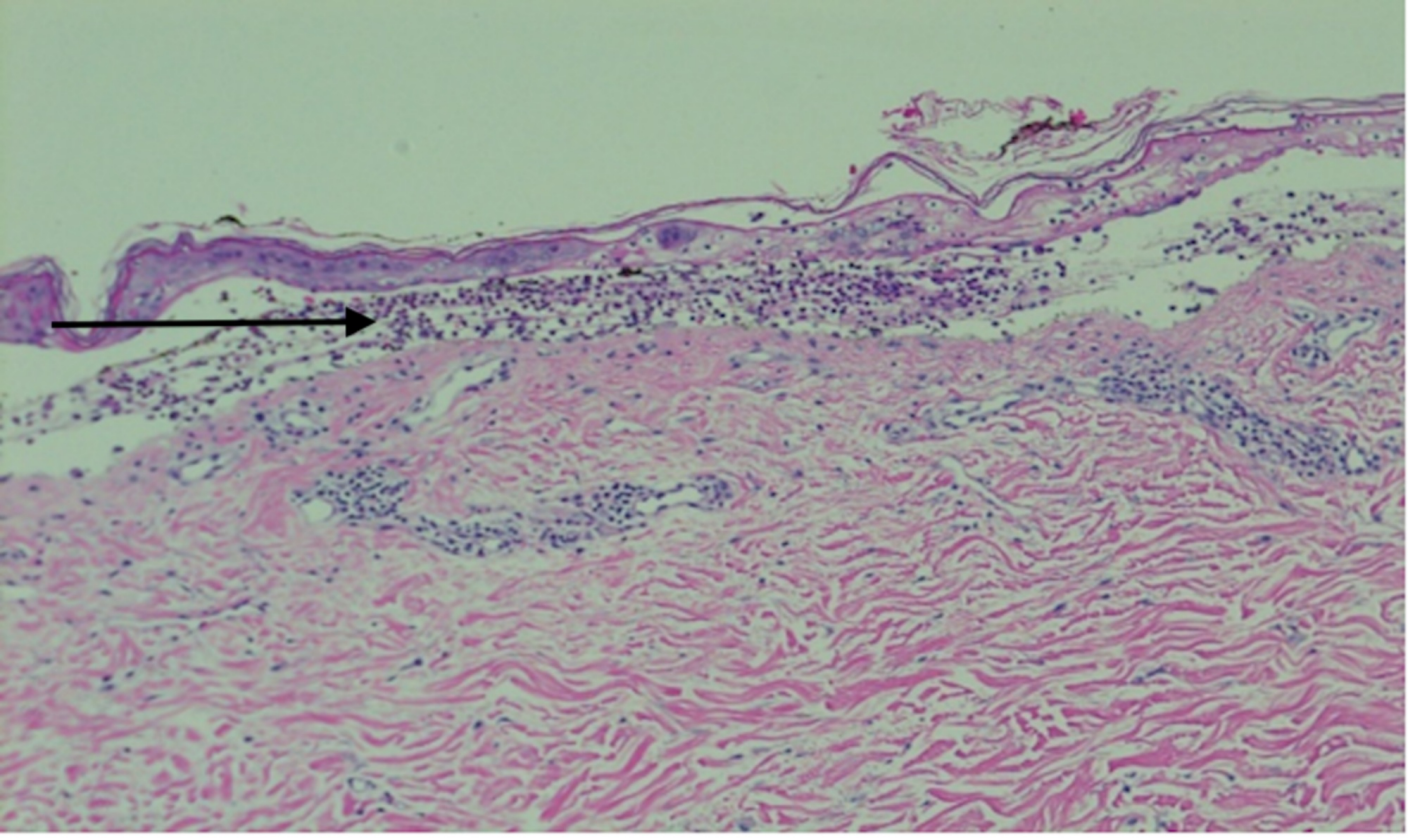
Hematoxylin and Eosin stain of the skin biopsy showing inflammatory cells mainly numerous neutrophils within subepidermal separation, as indicated by the arrow

He has no known history of drug allergies. Vital signs were significant for blood pressure of 111/76 mmHg, heart rate of 112 beats per minute, respiratory rate of 22 breaths per minute, temperature of 380 C and saturation of 89% on room air. Physical examination was significant for bilateral rhonchi on lung examination and approximately fifty 3-4 mm discrete, denuded vesicles and bullae on skin examination and quadriplegia. Laboratory work was significant for white blood cell (WBC) count of 6.5x109/L, hemoglobin of 8.9 g/dL, creatinine of 1.26, sodium of 152, potassium of 3.8; initial blood culture was negative, G-6-PD level was normal, urinalysis was suggestive of urinary traction infection but culture only grew candida species which was likely a contaminant given his chronic indwelling Foley catheter use. Chest X-ray and computed tomography (CT) of the chest were both suggestive of left lower lobe pneumonia. Echocardiogram showed preserved ejection fraction with no wall motion abnormalities. He was initially started on empiric valacyclovir, but skin swab came back negative for herpes and the medication was later discontinued. Vancomycin and piperacillin-tazobactam were empirically started for treatment of presumed health care-associated pneumonia. The last dose of vancomycin was given two days after admission. Five days later, he was found to have six to eight new blisters. A punch skin biopsy showed LABD (Figure 1). He was started on oral prednisone and dapsone. On the eighth day of admission, his rash started to improve with no new blistering. On day 13, he developed fever, tachycardia, and leukocytosis of 17x109/L. Extensively drug-resistant Pseudomonas aeruginosa was isolated in blood and urine, which were believed to be from superinfection of his skin lesions, and he was subsequently started on IV meropenem and tobramycin. Ultimately, he developed septic shock and the decision was made by his next of kin for hospice and comfort care. He passed away 11 days afterwards. 

## Discussion

Although drug-induced LABD is rare, its incidence has been increasing in frequency over the years. Clinicians should have a high index of suspicion for drug-induced LABD at the initial onset of vesicles and bullae after the administration of antibiotics. Multiple drugs have been reported to cause LABD, with vancomycin being the offending agent in 67% of cases [6]. While multiple studies have determined vancomycin to be the most common drug-induced cause of LABD, we found only one case reporting piperacillin-tazobactam inducing LABD in the absence of vancomycin co-administration. The case report by Adler et al. excluded vancomycin as the culprit drug by only administrating the antibiotic after the onset of the rash and documenting clinical improvement despite ongoing exposure [6]. Numerous reports mention drug-induced LABD but have little research conclusively proving a drug’s culpability. For instance, in one case study, 10 patients were on different drug regimens and were examined for LABD. It was concluded that vancomycin was the predominant culprit with lesser offending agents being piperacillin, tazobactam, ciprofloxacin, and trimethoprim-sulfamethoxazole [7]. Hence, we believe our patient's LABD was highly likely to be vancomycin induced than piperacillin-tazobactam, however, the patient’s hospital course also included the co-administration of piperacillin-tazobactam making the drug to potentially have a culprit effect. Early diagnosis correlates with decreased mortality and morbidity. Following the initial administration of vancomycin, LABD can occur anytime from one day to one month [8]. In our case, the patient received multiple doses of vancomycin and started to show signs two weeks after antibiotic administration. The diagnosis can be made by direct immunofluorescence searching for linear IgA deposition [9]. In our case report, punch biopsies of the lesions were taken instead confirming the LABD diagnosis (Figure 1).

Our patient represents a likely case of drug-induced LABD given the use of vancomycin and piperacillin-tazobactam. After strong suspicion of pneumonia, he was prescribed vancomycin and piperacillin-tazobactam. After 14 days of antibiotic treatment, he developed progressive, diffuse, tense rash with blisters that initially started over the scrotum. After three more days of vancomycin and piperacillin-tazobactam treatment, he was transferred to our hospital and continued to develop new blisters. Eventually, he was started on prednisone and dapsone resulting in short term improvement in his skin rash. Drug-induced LABD is a self-limiting disease and after discontinuing the offending agent, it may resolve spontaneously within a few days [10]. As in our case, the challenge of discontinuing medications is made difficult by patients being on multiple drug regimens. While literature supports vancomycin as the causative agent, we found few reports documenting piperacillin-tazobactam also inducing LABD. Clinicians are tasked with the decision of discontinuing treatment while balancing patient care. In severe cases, prednisone and/or dapsone should be given for 4-6 weeks [10]. In the literature, there are only a few case reports of vancomycin-induced LABD and most cases are resolved within a few days of discontinuing vancomycin along with supportive care. Unfortunately, in our case, the patient continued to receive multiple rounds of vancomycin before it was discontinued, and we believe that impacted his poor outcome.

## Conclusions

LABD not only mimics various other skin pathologies but also has a common variable presentation. Although drug-induced LABD has a low incidence rate, emerging case reports have shown strong evidence of clinicians overlooking adverse medication reactions. It is important to have a high index of suspicion for potential drug complications and halt poor outcomes by discontinuing the offending agent.
